# The Lung Microbiome in Idiopathic Pulmonary Fibrosis: A Promising Approach for Targeted Therapies

**DOI:** 10.3390/ijms18122735

**Published:** 2017-12-16

**Authors:** Aline Fastrès, Florence Felice, Elodie Roels, Catherine Moermans, Jean-Louis Corhay, Fabrice Bureau, Renaud Louis, Cécile Clercx, Julien Guiot

**Affiliations:** 1 Department of Clinical Sciences, FARAH, Faculty of Veterinary Medicine, University of Liège, 4000 Liège, Belgium; afastres@uliege.be (F.A.); eroels@uliege.be (R.E.); cclercx@uliege.be (C.C.); 2 Respiratory department, CHU Liège. Domaine universitaire du Sart Tilman, B35, 4000 Liège, Belgium; Florence.Felice@student.uliege.be (F.F.); c.moermans@chu.ulg.ac.be (M.C.); jlcorhay@ulg.ac.be (C.J.-L.), R.Louis@uliege.be (L.R.); 3 Laboratory of Cellular and Molecular Immunology, GIGA Research, University of Liège, 4000 Liège, Belgium; fabrice.bureau@uliege.be

**Keywords:** idiopathic pulmonary fibrosis, IPF, interstitial lung diseases, microbiome, microbiota

## Abstract

This review focuses on the role of the lung microbiome in idiopathic pulmonary fibrosis. Although historically considered sterile, bacterial communities have now been well documented in lungs both in healthy and pathological conditions. Studies in idiopathic pulmonary fibrosis (IPF) suggest that increased bacterial burden and/or abundance of potentially pathogenic bacteria may drive disease progression, acute exacerbations, and mortality. More recent work has highlighted the interaction between the lung microbiome and the innate immune system in IPF, strengthening the argument for the role of both host and environment interaction in disease pathogenesis. Existing published data suggesting that the lung microbiome may represent a therapeutic target, via antibiotic administration, immunization against pathogenic organisms, or treatment directed at gastroesophageal reflux. Taken altogether, published literature suggests that the lung microbiome might serve in the future as a prognostic biomarker, a therapeutic target, and/or provide an explanation for disease pathogenesis in IPF.

## 1. Introduction

Idiopathic pulmonary fibrosis (IPF) is a rare lung disease of unknown origin which leads rapidly to death [1,2]. However, the rate of progression of the disease varies among individuals and is still difficult to predict [3]. The prognosis of IPF is poor with a median survival of three to five years after diagnosis without curative therapies besides lung transplantation. However, two antifibrotic drugs, pirfenidone and nintedanib, are known to be effective in slowing down disease progression and in reducing lung related mortality [4,5]. The factors leading to disease initiation and progression remain incompletely known [1]. The current disease paradigm is that repetitive micro-injury to the alveolar epithelium by unknown environmental triggers (e.g., cigarette smoke, gastric microaspiration, particulate dust, viral infections or lung microbial composition) in genetically susceptible individuals leads to aberrant wound healing resulting in fibrosis rather than normal repair [6]. Numerous epidemiologic and genetic studies illustrate that genetic and environmental factors contribute to the risk of developing IPF [7]. In parallel to the different clinical phenotypes and genotypes discovered, molecular mechanisms promoting disease biology are also heterogeneous, and may involve an extensive array of different pathways and processes including apoptosis [8], oxidative stress [9], intra-alveolar coagulation [10], endoplasmic reticulum stress [9], and telomere shortening [11]. Previous studies have identified several genetic variants both associated with sporadic and familial forms of IPF that confer an individual predisposition to develop the disease [12,13]. Of interest, genes involved in host-bacterial defense, including alpha-defensin, have previously been described as up-regulated in IPF patients compared with control [14]. These studies suggest that genetic susceptibility in innate immune defense may play a role in the pathogenesis of IPF, and lend support to the concept that microbiota, through its interaction with the host immune system, may contribute to the sequence of events that result in fibrosis. Other reasons suggesting that infection by modulating microbial communities might interfere with fibrosis initiation or perpetuation processes are supported by the finding that immunosuppressive therapy decreases the progression-free survival time in IPF patients [6]. Besides these observations, adding antibiotics such as cotrimoxazole to specific anti-fibrotic therapies improves quality of life and reduces mortality [15]. Moreover, it is now widely recognized that despite the ancient dogma, the lungs are not sterile [16]. Culture-independent techniques have permitted to identify numerous micro-organisms coexisting in the lungs, such as bacteria, viruses and fungi [16,17]. This natural community of microorganisms, collectively known as the microbiome, populates our respiratory tract, and its role in healthy lung function is increasingly recognized. Not surprisingly, alterations to this respiratory microbiome are seen in multiple respiratory disorders. In the past few years, studies investigating the lower airway microbiome using these culture-independent techniques have shown an increased bacterial burden and taxonomic differences in IPF compared to healthy subjects [18,19]. Alterations of the microbiome may also drive disease progression or acute exacerbation [20]. While IPF microbiome studies have been able to derive bacterial genus and burden, they have not been able to establish a causal, mechanistic link to disease process or progression. It remains unclear whether the changes in lung microbiome reported in the IPF studies are a cause of the disease, or a consequence either of an underlying immune defense defect or of architectural changes. Therefore, recent studies aimed to investigate how the lung microbial community influences host defenses [21,22].

In this review we are going to approach successively the respiratory microbiome in healthy subjects and the main alterations described in IPF microbiome, during stable disease and during exacerbations, as well as its interaction with the host response. We have performed a systematic search in PubMed by typing the words: (“Microbiota”[Mesh]) and ((“Lung Diseases, Interstitial”[Mesh]) or “Idiopathic Pulmonary Fibrosis”[Mesh]) and (microbiome) and ((“Lung Diseases, Interstitial”[Mesh]) or “Idiopathic Pulmonary Fibrosis”[Mesh]), and selecting those thought to be relevant. Publications dates of selected papers range from 2010 to 2017. 

## 2. Microbiome in Healthy Lungs

The epithelial surfaces of the respiratory tract, previously thought to be sterile, have been shown, by using culture-independent techniques, to accommodate dynamic microbial communities. High-throughput bacterial 16s-rRNA sequencing has been described to identify bacterial DNA in 95.7% of bronchoalveolar lavage (BAL) specimens compared with conventional culture techniques, which detected bacteria in 39.1% of BAL samples [23]. 

The bacterial communities of healthy lungs closely resemble those of the mouth [24], while being two to four times lower in terms of bacterial burden. In lung tissues, a range from 10 to 100 bacterial cells per 1000 human cells has been previously reported [25]. It is also interesting to point out that despite differences in pH, temperature and oxygen concentration, the microbiome of healthy subjects is relatively constant between individuals [26,27]. The most four represented phyla in normal airways are Bacteroidetes (including the genus *Prevotella* sp.), Firmicutes (including the genera of *Streptococcus* sp. and *Veillonella* sp.) and, to a lesser extent, Proteobacteria and Actinobacteria [27,28,29].

The exact composition of the lung microbiota results from three main factors. The first one is microbial immigration due to microaspiration of gastric content, direct mucosal dispersion from the oro-nasal cavities, and to the inhalation of air. The second one is the microbial elimination, which results from the mucociliary clearance, cough and immunity. Finally, the third factor is the local microbial growth environment that includes notably nutrient availability, oxygen tension, pH and temperature. As a consequence of those three factors, the lung microbiota represents a steady state between microbial influx, efflux and reproduction rates, the latest being mostly altered in case of pathological processes. In every lung disease studied to date, the lung microbiome is altered compared with that of healthy subjects [30].

Given the sensitivity of the molecular technologies employed, an obvious concern in many studies is contamination of samples from the upper respiratory tract when sampling, providing a false representation of the true microbiome [18,19]. Although most of the published studies have characterized the lung microbiome of healthy subjects using BAL samples, the potential for oropharyngeal contamination should be addressed [31,32]. Besides, in healthy subjects, variation of the lung microbial community composition at spatially distinct lung sites within individuals have been shown [28], however, it remains lower than intersubject community variation [27]. It has recently been demonstrated that contamination contributes negligibly to microbial communities in bronchoscopically acquired specimens, validating the use of bronchoscopy to investigate the lung microbiome [32]. Bronchoscopy is not the only step where contamination can be introduced in studies of the microbiome. Significant variation has also been found when comparing microbiome data from the same patient samples using different sequencer platforms and methodologies [21,33]. Reagents and extraction kits are also significant sources of contamination and become particularly important with low biomass samples, like those generated from the respiratory tract [34,35]. Moreover, it must be remembered that sequencing DNA from a BAL sample provides a “snapshot” in time of the microbial diversity of the lower airways, but does not evaluate the dynamic changes that may be occurring longitudinally. 

Beyond studying the microbiota, a small minority of studies have focused on fungi and viruses. Recent studies have shown that commensal fungi not only affect the host immune system, but can also affect bacterial composition and have a particularly important influence during restoration of the bacterial microbiota after antibiotic treatment [36]. Virome is known to be highly variable in lungs and is thought to be a trigger in multiple lung diseases [37].

## 3. Microbiota in IPF (Idiopathic Pulmonary Fibrosis)

Even if the exact pathophysiology of IPF is still incompletely understood, the microbiome is suspected to play a role in the pathology [38]. Indeed, bacteria can cause epithelial alveolar injury on their own, but can also activate an immune cascade response due to their presence alone [39], the following pro-inflammatory and pro-fibrotic cascades resulting in alterations of the lung architecture.

The hypothesis that IPF progression is influenced by microbes is supported by the finding that immunosuppression increased the risk of death and hospitalization [6]. Another clue concerning the role of bacteria in IPF pathogenesis is the effect of antibiotics on the natural history of the disease. Sulgina and al. attempted to treat 181 IPF patients with cotrimoxazole for 12 months [15], and showed a decreased mortality rate and an increased quality of life, with a decreased need of oxygenotherapy, however, it did not translate into an improvement of pulmonary function. Respiratory infections were also less frequent among the treated group. Unfortunately, almost one-third of patients receiving cotrimoxazole withdrew from the trial due to side effects, mostly rash and nausea. 

The microbiome of IPF patients is distinct from healthy individuals: their bacterial load is overall higher, and the genera *Haemophilus*, *Streptococcus*, *Neisseiria* and *Veillonella* sp. are more abundant in patients with IPF compared to controls [40]. It is also very different from the mouth microbiome in comparison to healthy subjects, which suggests a microbial selection in the lower respiratory tract in chronic lung disease [27], as each disease seems to have its own microbial signature, including a loss of diversity along with dysbiosis [41].

The first exploratory application of a culture-independent molecular technique in IPF studied the microbiome in BAL from 17 IPF patients [42]. Using 16s-rRNA gene polymerase chain reaction (PCR) and degenerating gel electrophoresis (DGGE), the study found organisms often associated with the oropharynx, as well as uncultured bacterial sequences corresponding to the *Streptococcus*, *Neisseria* and *Actinobacterium* sp. genera. Interestingly, bacterial DNA was not detected in five out of eight patients colonized with *Pneumocystis jirovecii*, suggesting this fungus may impair bacterial colonization of the airways [42]. 

A small study then investigated the upper and lower respiratory tract microbiota in a heterogenous group of 18 patients with interstitial lung disease (ILD), including five with idiopathic interstitial pneumonia (IIP), six patients with pneumocystis associated pneumonia and nine healthy controls [43]. The 16s-rRNA gene sequencing of BAL revealed no significant differences in the microbiome between ILD and healthy controls. There was a signal toward lower bacterial diversity in the IIPs but this was not statistically significant. 

Later on, a multicenter cohort study of Correlating Outcomes with biochemical Markers to Estimate Time-progression in idiopathic pulmonary fibrosis (COMET) [19], retrospectively characterized the lung microbiota in 55 IPF patients with no active infection at the time of screening by sequencing the genome of the bacteria found in baseline bronchoalveolar lavage fluid (BALF) samples. The study also followed-up participants prospectively at 16 weeks intervals up to 80 weeks in order to provide longitudinal outcome data. In that study, the most prevalent OTUs (operational taxonomic unit) in IPF patients were *Prevotella*, *Veillonella* and *Cronobacter* sp. Moreover, the presence of a specific *Streptococcus* or *Staphylococcus* OTU (among all *Streptococcus* and *Staphylococcus* OTUs) above a certain threshold was associated with a faster-progressing disease, after adjusting for age, sex, smoking status, respiratory function, six-minute-walk test and the presence of gastro-intestinal reflux [19]. However, those OTUs were only found in less than half of the IPF cohort; their presence is thus insufficient to explain the disease pathogenesis on its own but these findings open the possibilities to use those OTUs as prognostic biomarker for disease progression. A limitation to this study is that 16S rRNA sequencing could not be used for species-level identification. Further work, in the form of either culture-specific or microbe-specific sequencing, is needed to formally identify these bacteria. 

A large study published in 2014 investigated 65 well-defined IPF patients and 44 controls which included 27 healthy controls and 17 patients with moderate chronic obstructive pulmonary disease (COPD) [18]. The first notable finding was a twofold higher bacterial load (quantified by 16S rRNA gene/mL BALF) in IPF BALF compared with control subjects (*p* < 0.0001). Secondly, there was a significant association between patients with higher BALF bacterial load and disease progression at six months (defined by a decline in forced vital capacity (FVC) by 10%, or death) compared with controls (*p* = 0.02). Furthermore, it was possible to stratify patients according to bacterial burden in order to predict mortality risk, patients with higher bacterial burden having an increased risk of mortality (hazard ratio: 4.59) compared with patients with low bacterial burden. After logistic regression analysis, the abundance of *Veillonella*, *Neisseria*, *Streptococcus* and *Haemophilus* sp. remained significantly associated with IPF. Moreover, the study found that patients carrying a minor allele at the MUC5B promoter had a lower bacterial burden, providing a mechanistic link between bacterial burden and a mutation known to be relevant in IPF. 

## 4. Host Microbial Interactions in IPF

Today, it is still unclear whether changes in microbiome seen in IPF are a cause or a consequence of the disease. The PANTHER trial [6] has identified an increase in mortality with immunosuppressive therapies, suggesting a role of a deficient host immunity in the pathogenesis of the disease. In addition, acute infection is also associated with a greater mortality rate in IPF patients, highlighting once more the crucial role of the immune system in the natural history of the disease [44]. Consequently, studies investigating whether the lung microbiome influences the host defense in IPF are needed. Search for associations between alterations in the lung microbial community in IPF and host immune response in IPF have been addressed in recent publications [21,22]. 

In a study from Molyneaux et al. [21], the authors investigated a cohort of 60 patients with IPF from the Interstitial Lung Disease Unit at the Royal Brompton Hospital, London, and 20 matched healthy controls. All participants underwent BAL and blood sample collection. In IPF patients, BAL was performed at baseline. Moreover, for the longitudinal follow-up of these patients, peripheral blood samples were obtained and pulmonary function was further tested up to 12 months after diagnosis. Researchers analyzed gene expression of the host and found two particular groups of genes whose expression correlated with an IPF diagnosis, with higher bacterial burden in the BALF, and specific OTUs. The genes identified also correlated with an increase of neutrophils in both BAL and blood samples. These groups included genes involved in host defense response (*Nlrc4*, *Pglyrp1*, *Mmp9*, *Defa4*). Additionally, that team found two genes encoding specific antimicrobial peptides (*Slpi* and *Camp*). Several of the identified genes were linked to poor survival and disease progression. These results suggest a host response to alterations of the respiratory microbiome in IPF, suggesting that these microbial changes would possibly trigger a response associated with the damage often observed in IPF patients. As they conclude, “the bacterial communities of the lower airways may act as persistent stimuli for repetitive alveolar injury in IPF” [21].

Another independent study [22] evaluated peripheral blood mononuclear cell (PBMC) gene expression, BALF microbiome and in vitro fibroblast responsiveness to cytosine-phosphate-guanine (CpG) antigenic stimulus in 68 IPF patients. Relative inhibition of several gene signaling pathways was associated with reduced progression-free survival time (PFS); some pathways were involved in immune inflammatory response and pathogen infection-like regulation of autophagy, while others are involved in pattern recognition receptors such as Toll-like receptor signaling pathway for example. The down-regulation of immune response pathways is associated with modifications in the abundance of specific OTUs. Indeed, they showed that the decrease of nucleotide binding oligomerization domain (NOD)-like receptor signaling is associated with increased abundance of *Streptococcus* sp. OTU, and that this phenomenon is correlated with poorer PFS. *Staphylococcus* and *Prevotella* sp. OTUs are also associated with poorer PFS, with decreased expression of immune response pathways genes and with overexpression of TLR-9 in PBMC. Finally, the increased presence of a specific *Veillonella* sp. OTU is correlated with increased CpG fibroblast responsiveness. This study demonstrates that host defense, as assessed by immune pathway gene expression, may be modulated by variations in the lower airway microbiome and that bacteria with increased abundance and decreased diversity are associated with decreased immune pathway genes expression and poorer PFS. This study also demonstrates that host-microbiome interaction may influence immune-mediated fibroblast responsiveness.

On the other hand, Wang et al. [45] have attempted to treat IPF patients with aerosolized interferon-gamma as a single therapy. The diversity of the microbiome was not impacted by the treatment; however, the study established a connection between the composition of the microbiome and the disease phenotype regarding inflammatory and fibrotic markers in the lung mucosa, suggesting once more an interaction between host immunity and microbiome.

## 5. Microbiome Effect on IPF Prognosis and Exacerbation

The progression of IPF is marked with exacerbations, similar to a number of chronic lung diseases. Acute exacerbations are associated with a particularly poor prognosis. Among patients with acute exacerbations, non-survivors had shorter durations of dyspnea, lower arterial oxygen tension (PaO_2_)/inspiratory oxygen fraction (FiO_2_) ratios, higher C reactive protein (CRP) levels, higher percentages of neutrophils and lower percentages of lymphocytes in BALF compared with survivors. Amongst those factors only CRP was found to be an independent predictor of survival, suggesting that infection (either bacterial or viral) and/or inflammation can be one of the pathogenic mechanisms contributing to acute exacerbations [46].

An exacerbation is currently defined as “an acute, clinically significant deterioration of unidentifiable cause in a patient with underlying IPF” [44], and it requires formal exclusion of an infection for clinical diagnosis. However, the exact pathogenesis of acute exacerbations remains unknown, and it is currently unclear whether it represents an accelerated phase of an underlying fibroproliferative process or an exaggerated lung injury response to unidentified preceding or coexistent infection [20]. Factors supporting a role for infection in exacerbation include the fact that respiratory tract infections in individuals with IPF confer a mortality risk indistinguishable from that seen with acute exacerbations. Moreover, the definition has been challenged by recent studies of the lung microbiome during IPF exacerbations and its influence on disease progression [18,20]. Such studies show that a high bacterial burden at the time of diagnosis seem to be a biomarker for a more-rapidly progressive disease with an increased risk of mortality [18].

Another study on 20 patients with diagnosed acute exacerbations of IPF and 15 matched controls with stable IPF who underwent bronchoscopy and DNA extraction has shown that IPF patients presented an increased bacterial burden during exacerbations, up to four times higher [20]. Their BALF also contained more neutrophils compared to stable IPF patients. This raises the possibility of bacteria playing a role in exacerbations, regardless of the presence of an active infection. They also analyze 16S rRNA gene qPCR and pyrosequencing in both stable and acute exacerbation group in order to explore changes in the BAL microbiota. In case of acute exacerbation there was a notable change in the microbiota with an increase in two potentially pathogenic Proteobacteria OTUs, *Campylobacter* sp. and *Stenotrophomonas* sp., coupled with a significant decrease in *Veillonella* sp., and *Campylobacter* sp., although best known as a gastrointestinal pathogen, was previously identified in the respiratory microbiota of individuals with severe COPD. Its presence in the respiratory microbiota is likely to arise from silent micro-aspiration of gastric contents [20]. To summarize these observations, this pilot study suggests that bacteria may play a causative role in acute exacerbation of IPF. The apparent translocation of bacteria usually confined to the gastrointestinal tract also suggests a role for micro-aspiration. Results of this study, although requiring a prospective longitudinal study for validation, provide a rationale for clinical trials of prophylactic antibiotics as a strategy to prevent acute exacerbations in individuals with IPF.

## 6. Conclusions and Perspectives

All of these findings open the possibility of a place for antibiotherapy in IPF patients; in particular, they provide a rationale for clinical trials of long-term antibiotherapy acting as an immunomodulator and an antibioprophylaxis to prevent acute exacerbations. In the future, an improved knowledge of the dynamic alterations of the lung microbiome might help to select appropriate, targeted and more personalized antibiotherapy in the course of the disease, in particular in IPF exacerbations. In this context, more advanced metagenomic analyses are required to elucidate the functional role of individual bacterial genera and communities in IPF progression.

As recent studies demonstrated interactions between host immune response and microbial community in IPF, further studies will probably focus on the exploration of therapeutic approaches targeting modulation, not only of the lung microbial community of patients with IPF, but of specific components of the innate immune system.

## Abbreviations

IPF	Idiopathic pulmonary fibrosis
BAL	Bronchoalveolar lavage
DGGE	Degenerative gel electrophoresis
PCR	Polymerase chain reaction
ILD	Interstitial lung disease
IIP	Idiopathic interstitial pneumonia
COMET	Correlating outcomes with biochemical markers to estimate time-progression
BALF	Bronchoalveolar lavage fluid
OTU	Operational taxonomic unit
COPD FVC	Chronic obstructive pulmonary disease Forced vital capacity
PBMC CpG	Peripheral blood mononuclear cell Cytosine-phosphate-guanine
PFS	Progression-free survival time
NOD PaO_2_ FiO_2_	Nucleotide binding oligomerization domain Lower arterial oxygen tension Inspiratory oxygen fraction
CRP	C-reactive protein
